# Impact of Small Area Level Deprivation on Colorectal Cancer Survival: Findings from the Regional Cancer Registry in Korea

**DOI:** 10.3390/cancers17040567

**Published:** 2025-02-07

**Authors:** Seung Min Hong, Ahreum Kim, Changhoon Kim, Seunghye Jang, Dong Uk Kim, Dong Hoon Baek, Seung Hun Lee, Yu Hyeon Yi, Heeseung Park, Jonghyun Lee, Tae In Kim, Hyun Joo Lee

**Affiliations:** 1 Department of Internal Medicine, Pusan National University School of Medicine, Busan 49241, Republic of Korea; lucky77i@naver.com (S.M.H.); dhbeak77@gmail.com (D.H.B.); keiasikr@nate.com (J.L.); zeitgeister88@daum.net (T.I.K.); 2 Division of Gastroenterology, Department of Internal Medicine, Pusan National University Hospital, Busan 49241, Republic of Korea; 3 Biomedical Research Institute, Pusan National University Hospital, Busan 49241, Republic of Korea; wkdtmdgpwkd@naver.com (S.J.); greatseunghun@daum.net (S.H.L.); eeugus@hanmail.net (Y.H.Y.); paul0109@naver.com (H.P.); atouchofbreeze@gmail.com (H.J.L.); 4 Office of Public Healthcare Service, Pusan National University Hospital, Busan 49241, Republic of Korea; ar_kim_@naver.com; 5 Department of Preventive Medicine, Pusan National University School of Medicine, Busan 49241, Republic of Korea; 6 Division of Gastroenterology, Department of Internal Medicine, CHA University Gumi Medical Center, Gumi 39295, Republic of Korea; amlm3@hanmail.net; 7 Department of Family Medicine, Pusan National University School of Medicine, Busan 49241, Republic of Korea; 8 Department of Family Medicine, Pusan National University Hospital, Busan 49241, Republic of Korea; 9 Department of Surgery, Pusan National University School of Medicine, Busan 49241, Republic of Korea; 10 Department of Surgery, Pusan National University Hospital, Busan 49241, Republic of Korea; 11 Department of Obstetrics and Gynecology, Pusan National University School of Medicine, Busan 49241, Republic of Korea

**Keywords:** colorectal cancer, deprivation index, survival

## Abstract

Background/Objectives: Research on the relationship between small-area-level deprivation and cancer survival, particularly for colorectal cancer (CRC), is lacking. Therefore, we investigated the relationship among small area-level deprivation, individual-level factors, and CRC survival using data from the Busan Regional Cancer Registry. Methods: We analyzed 34,999 patients with CRC from the Busan Regional Cancer Registry from 2003 to 2020. The primary outcome was CRC mortality. The explanatory variables at the individual level included age, gender, cancer stage, and year of diagnosis, whereas the Deprivation Index (DI) was used at the regional level. We conducted a multilevel survival analysis with frailty to assess the impact of individual- and area-level factors on survival probabilities. Results: In the multilevel survival model, each unit increase in the DI at the area level was associated with a 6.6% decrease in survival probability. When applying Model 2 and deriving regional estimates using the empirical Bayesian estimation method, the graph of the DI (x-axis) against survival probability (y-axis) showed that the slope of the regional DI for the 3-year and 5-year survival probabilities increased compared with the 1-year rate across all stages of the disease. Additionally, the slopes were steeper for the distant stage than for the local or regional stages. Conclusions: Small-area level deprivation negatively affects CRC survival, especially in distant-stage patients and those with longer disease duration.

## Simple Summary

This study examines the impact of socioeconomic deprivation at the small-area level on colorectal cancer (CRC) survival. By analyzing data from 34,999 CRC patients in the Busan Regional Cancer Registry, we explored survival disparities linked to individual and regional factors. Our findings indicate that greater deprivation correlates with lower survival probabilities, particularly among patients with advanced-stage disease and over longer periods. This research highlights the importance of addressing socioeconomic inequalities in health policy and cancer care and advocates for targeted measures to improve treatment accessibility in deprived areas. The insights gained can inform health equity initiatives and resource allocation to enhance CRC survival outcomes.

## 1. Introduction

In 2020, colorectal cancer (CRC) was the third most common cancer in terms of incidence, death, and prevalence in South Korea [1] and has been designated as one of the five major cancers (gastric cancer, hepatocellular carcinoma, CRC, breast cancer, and cervical cancer) for national management through the National Cancer Screening Program (NCSP). Health equity is defined as the absence of unnecessary, avoidable, unfair, and unjust health differences [2] and is recognized as a crucial element in developing healthcare systems in many countries. The Korean government has consistently included the promotion of health equity in its goals in the Korean Health Plan [3,4,5,6]. However, health equity is influenced by socioeconomic, racial, and geographical factors. Previous studies have reported disparities in the incidence, survival, and mortality of various cancers based on socioeconomic groups, regions, and races [7,8]. One study using data from the 2009 Korean National Health Insurance Service (K-NHIS) cancer registration analyzed the relationship between family income class and cancer risk and reported a higher cancer risk in the lowest income class than in the highest income class [9]. Another study utilizing data from the 2018 Korea Central Cancer Registry (KCCR) reported that higher-income groups had lower incidence rates of colorectal and cervical cancers, whereas the incidence rates of prostate and thyroid cancer were higher [10]. Additionally, social deprivation also affects cancer screening. Clarke et al. reported a significant correlation between the Deprivation Index (DI) and fecal immunochemical tests of CRC screening uptake [11]. Similarly, Smith et al. found that socioeconomically deprived women were less likely to participate in breast cancer screening [12]. These indicators related to cancer are influenced by various socioeconomic factors and levels of deprivation.

The area is a significant factor in the prevention and management of cancer. Studies conducted in some high-income countries have identified the association between area-level deprivation and social risk factors (such as poor housing conditions, high unemployment, regional migration rates, low household income, high inequality, and a composite index of poverty) and cancer, using traditional ecological analysis and the geographic information system (GIS) [13,14,15]. Specifically, some research implemented an area-based approach in Germany (GISD, German Index of Socioeconomic Deprivation) and investigated socioeconomic inequalities in cancer incidence and mortality. GISD is a composite index with three classic socioeconomic dimensions (income, education, and employment), with a higher index score indicating increased socioeconomic deprivation [16]. Notably, socioeconomic inequalities in the incidence and mortality of common cancers present opportunities for population-based cancer prevention [16] and can highlight regions with substantial needs for cancer prevention and control across Germany [17]. Moreover, area-based cancer inequalities can identify districts with elevated rates of specific cancers, facilitating the development of local and community-based strategies for cancer prevention and control [16]. In addition, because area-level factors influence health through material infrastructure and collective social functions, independent of the compositional characteristics of residents, their proper operationalization enables testing causal hypotheses linking area-level factors to health outcomes [18]. Furthermore, innovative approaches to cancer diagnosis and treatment, including artificial intelligence and molecular biology-based techniques, continue to be developed [19,20,21]. However, social and structural factors can serve as barriers, limiting patients’ access to these advanced diagnostics technologies [22]. A previous study analyzing diagnostic pathways for major cancers revealed that patients with greater deprivation were more likely to be diagnosed incidentally or symptomatically rather than through regular checkups. These patients were also more frequently diagnosed at distant stages of the disease [23]. This suggests that deprivation hinders access to diagnostic testing, ultimately leading to diagnoses at more advanced stages of cancer progression.

In South Korea, many studies on health equity have focused on the relationship between socioeconomic status, deprivation, and health status. However, few studies have used national registry data to explore the relationship between cancer survival and small area-level deprivation [10]. To our knowledge, no study has investigated the relationship between survival and small-area-level deprivation specifically for CRC.

Importantly, Busan exhibits the highest cancer mortality rate among the six metropolitan cities in South Korea, which may be due to the higher level of health inequality in Busan compared to that of other regions [24]. In fact, Busan has the highest composite regional deprivation index among all metropolitan and provincial areas in South Korea [25], highlighting the alleviation of health inequalities as one of the most critical challenges for improving the overall health status in the region. To address this, a detailed analysis of small-area level deprivation in Busan is necessary. This study used data from the Busan Regional Cancer Registry to examine the relationship between area-level deprivation, individual-level factors, and CRC survival in Busan, considering the health policy implications of these findings.

## 2. Materials and Methods

### 2.1. Data Source

The dataset used in this study was the Busan Regional Cancer Registry, which collects cancer incidence data from Busan. It is part of the KCCR, which collects nationwide cancer incidence data [26]. The completeness of cancer registration in Korea is estimated to be 97.8% [27]. This study focused on CRC data from the entire Busan population (*n* = 34,999) from 2003 to 2020. We linked national mortality data to identify the date of death and mortality from CRC and other causes. The follow-up period began on the date of diagnosis and ended on 31 December 2022.

### 2.2. Method

#### 2.2.1. Outcome Variable

The outcome variable was CRC mortality, measured as the time from diagnosis to death or the end of the study period. The Busan Regional Cancer Registry was used to identify primary diagnoses of CRC in the study population. Only primary colon neoplasms classified according to the International Classification of Diseases 10th Revision (ICD-10) were studied. CRC mortality (ICD-10 = C18-C20) was identified in the Cause of Death Registry during the same period.

#### 2.2.2. Explanatory Variables

At the individual level, explanatory variables included age groups divided into four lifecycles (0–44, 45–64, 65–74, ≥75), gender, stage (local, regional, and distant), and year. This age classification is similar to the one newly proposed in the recent colorectal cancer screening guidelines [28]. At the regional level, it was divided into 205 neighborhoods based on individuals’ places of residence. The regional unit is the smallest administrative unit for the administrative and social services provided by administrative welfare centers. The explanatory variable at the regional socioeconomic level was the DI. The DI was calculated using the method described in previous studies [29]. It was based on the 10% sample data from the 2015 Population and Housing Census with socioeconomic conditions like housing environment, education, social class, senior population, single-person households, households without cars, non-apartment housing, female-headed households, divorces, and separated households. Principal components and factor analyses with varimax rotation were applied to summarize these data. The DI was constructed by summing up the Z-standardized scores of individual variables to have a population-weighted mean of 0 and a variance of 1. Higher index values indicate higher deprivation levels, i.e., lower socioeconomic position of a district’s population. All regions included in our analysis were classified into quintiles of socioeconomic deprivation by 2015 according to their DI values.

#### 2.2.3. Statistical Analysis

**Figure 1:**
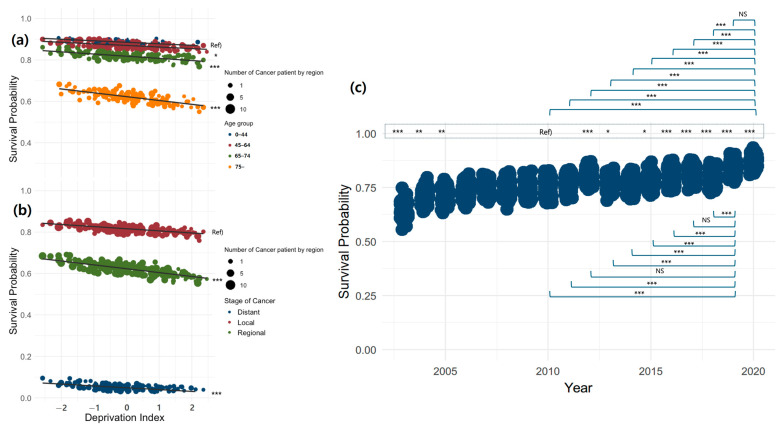
The difference in the estimated marginal means for each factor that showed a significant difference in Model 2, where (**a**) visualizes the difference by age group, (**b**) by stage at diagnosis, and (**c**) by the time of diagnosis (*: <0.05, **: <0.01, ***: < 0.001). NS: not significant.

**Figure 2:**
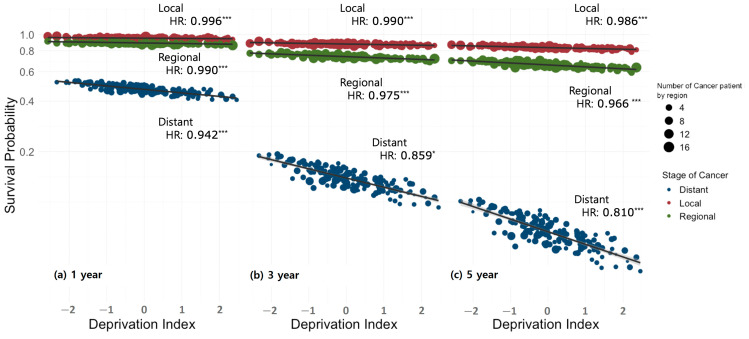
The association between stage at diagnosis and deprivation level on 1-, 3-, and 5-year area-level survival probability (*: <0.05, ***: < 0.001). HR: hazard ratio.

Using the Kaplan–Meier method, the median survival time was calculated for each individual factor that affected survival, and comparisons between groups were made using the log-rank test. For subjects residing in the same region, a multilevel structure with the same DI was used, and a multilevel survival analysis with frailty was conducted to examine individual and regional factors related to CRC survival. Statistical significance was assumed for a two-tailed *p*-value less than 0.05. To evaluate the relationship between individual-level risk factors and regional-level deprivation, a series of models were examined, starting from simple models to complex models, by adding individual- and regional-level predictor variables. First, to examine regional variation, a model with only a random intercept (random effect-only) was estimated, and then individual characteristics were included in the model to investigate the extent to which regional variation is explained by differences in individual composition. The calculated hazard ratio (HR) represents an increased risk of occurrence in a specific category compared with the reference category if the value is greater than 1. Next, to investigate whether regional variation was conditioned by regional characteristics, the level of regional DI was added to examine the explanatory power of regional-level variation [30,31]. To directly compare the HR of individual variables with variations at the regional level, the median hazard ratio (MHR), which quantifies group heterogeneity (cluster effect), was presented. We applied the empirical Bayesian estimation method to our analysis model to derive area-level estimates. The results are presented in Figure 1, which shows the marginal effect estimation test for each factor. To provide a clear picture of the DI slope, we opted for a log transformation of the y-axis of the survival probability, making it easier to understand the HR, as shown in Figure 2.

#### 2.2.4. Ethical Considerations

This study analyzed existing data from the Busan Regional Cancer Registry with no patient intervention. No identifiers were linked to the individuals. This study was approved by the Pusan National University Hospital Institutional Review Board (IRB) under the category “exempt” status (IRB ID: H-1412-012-024).

## 3. Results

From 2003 to 2020, we analyzed 34,999 patients with CRC from the Busan registry. Table 1 presents the median survival probability of patients with CRC according to study characteristics. Most patients were 45–65 years old (44.5%, *n* = 15,577), men (60.0%, *n* = 21,006), and regional (45.3%, *n* = 15,872). A significant increase in cancer diagnoses was observed from 2003 to 2020 (*p* < 0.001). The least deprived group comprised 20.5% of all patients with CRC, and the most deprived group comprised 19.2%. This proportion generally decreased as deprivation increased (*p* < 0.001).

Examination of the survival probability of patients with CRC demonstrated significant variations across various study parameters. The 0–44 age group demonstrated the highest survival probability (85.4%), while individuals aged 75 years and above exhibited the lowest survival probability (59.4%). Gender-based survival probabilities showed minimal disparity, with men presenting a marginally higher median survival probability of 78.6% than women (76.5%). Regarding disease stage, the local group displayed a superior median survival of 92.3%, whereas the regional and distant groups showed 82.8% and 31.5%, respectively, highlighting significant stage-dependent differences. Temporal analysis indicated an improvement in survival probabilities, from 68.0% in 2003 to 87.5% in 2020. Conversely, survival probabilities declined with increasing DIs, ranging from 80.5% in the least deprived areas to 74.9% in the most deprived areas. The overall median survival duration was 157 weeks.

Table 2 presents the fixed and random parameters estimated using the frailty (multilevel) survival model. Significant area-level differences were observed, as the confidence interval of the MHR for the survival probability did not include 1, from the random effect-only model (Model 0) to the model that included individual and area-level variables (Model 2). The degree of area-level heterogeneity tended to decrease when individual and area-level variables were included. The inclusion of individual-level variables (Model 1) revealed statistically significant associations across all variables, consistent with the findings shown in Table 1. Notably, the area-level variation decreased by 6%, suggesting that the composition of individual-level variables within areas explained this portion of the regional variation. Subsequently, incorporating the DI (Model 2) led to a further 7% reduction in area-level variation, highlighting the index’s significance in elucidating regional disparities. Analysis of DI, an area-level variable, revealed a significant impact on survival probabilities. The index, ranging from −2.56 to 2.49, demonstrated a 6.6% decrease in survival probability for each unit increase. This translates to a potential 33% difference in the survival probabilities across the index’s range, an effect comparable to the impact of an aging population. In Model 2, which incorporated DI, the parameters for age and stage at diagnosis decreased slightly compared with Model 1 while maintaining their overall direction and significance. Gender and year of diagnosis remained largely unchanged. This suggests that Model 2 effectively controlled for the confounding effects of DI, age, and stage at diagnosis, providing a more comprehensive analysis of survival determinants. Following the proportional hazards assumption, Model 2 shows distinct survival curves for different hazard levels that do not intersect. Notably, these curves display widening gaps over time. The analysis revealed a 5-year survival rate of approximately 60%, adjusted for specific individual and regional variables. Moreover, regional-level random effects showed statistical significance (see Appendix A).

Figure 1 and Figure 2 apply Model 2 and use the empirical Bayesian estimation method to derive regional estimates. Point estimates are presented for statistically significant variables by region; however, errors for individual regions are not provided, with the objective being to avoid overloading figures and facilitate the identification of trends. Figure 1 shows the difference in the estimated marginal means for each factor that showed a significant difference in Model 2, where (a) visualizes the difference by age group, (b) by stage at diagnosis, and (c) by time of diagnosis. This pattern is the same as that presented in Table 2. The survival probability was lower in the older age group than in the 0–44 age group and in the regional and distant groups than in the local group. Regarding the time of diagnosis, the survival probability was lower in 2003–2005 than in 2010, and, since 2012, all patients showed a higher survival probability, except in 2014. In 2020, the most recent year, there was no difference from 2019, but it had the highest survival probability since 2010, and 2019 showed a higher survival probability than did all other years, except 2017 and 2012. Considering annual variations, the data indicate an upward trend in the survival probability since 2018.

Figure 2 shows the effects of the stage at diagnosis and deprivation level on the 1-, 3-, and 5-year survival probabilities. Due to the characteristics of the model, where the survival probability decreases by an exponent of time as time passes, and the proportional hazard assumption, the slope of the regional DI for 3-year and 5-year survival probabilities increased compared to the 1-year survival probabilities in all stages of the disease. Additionally, the slopes were steeper for the distant stage than for the local or regional stages.

## 4. Discussion

This study analyzed data on CRC survival in the Busan region using a multilevel survival analysis. Our findings indicated a negative correlation between deprivation levels and CRC survival probabilities. The regional cancer registry provided a substantial dataset that enhanced the statistical reliability of our results. To the best of our knowledge, this is the first study to investigate the relationship between CRC survival and small area-level deprivation. Furthermore, our analysis revealed that the impact of deprivation on cancer survival intensifies as the disease progresses to advanced stages.

Previous studies have examined the survival rates of various cancers in relation to the DI. A recent study utilizing the KCCR database reported that a higher DI is associated with an increased mortality risk among patients diagnosed with six cancer types, including stomach, colorectal, liver, breast, cervical, and lung cancers [32]. However, this study did not calculate DI at a granular geographic level as our research did. Similarly, a relatively recent study by Hufnagel et al. found that although state- and national-level area deprivation indices were not associated with cancer characteristics, they negatively impacted mortality risk [33]. In 2011, research targeting the Busan region computed the deprivation index at the town level and demonstrated its association with mortality from various diseases; nonetheless, it did not provide detailed analyses by cancer type [29]. Unlike previous studies, we calculated DI at the small-area level and investigated its relationship with various indicators of colorectal cancer. The finding that deprivation has a negative impact on survival is largely consistent between our study and earlier research. However, we provided a more in-depth analysis of how individual factors and disease statuses interact with socioeconomic factors to influence the survival of colorectal cancer patients.

In our study, the number of patients with CRC exhibited a notable trend, with the lowest count recorded in 2003 (828 patients) and peaking in 2013 (2439 patients, 7.0%). After 2013, a slight decline was observed, but the rate stabilized at around 6–7% by 2020. The significant increase in the number of CRC cases during the initial study period can be attributed to the implementation of Korea’s national CRC screening program in 2004. The national cancer screening participation rate in Korea has grown substantially, rising from 19.9% in 2004 to 60.1% in 2014. Subsequently, this rate maintained relative stability for approximately 5 years without significant further increases [34]. Additionally, the increase in the incidence of CRC is related to the number of colonoscopy procedures performed. Kim et al. reported that the incidence of invasive CRC increased as the number of colonoscopy procedures increased, based on an analysis of the K-NHIS claims data from 2002 to 2020 [35]. The increase in early-onset CRC (EOCRC) in younger age groups is also thought to have contributed to the increase in the number of patients with CRC. Several studies have recently reported an increase in the incidence of EOCRC. A large-scale study using the United States Cancer Statistics Database showed an overall increase in EOCRC incidence in the United States between 2001 and 2016 [36]. The increase in EOCRC is a global trend observed not only in the United States but also in Europe, Iran, Egypt, and Australia. One study reported that the increase in the incidence rate of CRC in patients under the age of 50 years was higher than that in patients over 50 years in the United States, United Kingdom, Australia, Canada, Brazil, Shanghai, Japan, and Hong Kong [37,38,39,40]. In Korea, the incidence of EOCRC steadily increased from the 1990s until 2011 and has remained relatively stable since early 2011 [41]. The slight decrease in the number of patients after the sharp increase in the early study period is thought to be due to the high accessibility of medical services in Korea, leading to frequent colonoscopies and polypectomies even in younger age groups [42].

When individual-level variables were added to the random effect-only analysis model, the survival probability was higher for younger age, female gender, and locoregional stage. It is well known that survival probability decreases significantly in cases of distant metastasis compared to locoregional stages, as curative surgical resection is not possible, except for some resectable metastases. In Korea, the 5-year relative survival probability of patients with CRC from 2015 to 2019 showed a marked difference between the localized stage (73.4%) and distant metastasis stage (24.4%) [34]. In our study, the HR for the regional stage was 2.325 (95% confidence interval [CI] 2.166–2.497), and, for the distant stage, it was 15.09 (95% CI 14.07–16.19). These results indicate that the stage at diagnosis is one of the most significant factors negatively impacting cancer patient survival. Evidence suggests that socioeconomic factors can influence the stage at which CRC is diagnosed. A comprehensive population-based study revealed a significant disparity in mortality rates between the most deprived and affluent areas among male patients with CRC. The study found a difference of 131 deaths per 1000 person-years, with 42 of these deaths attributable to variations in the stage at diagnosis [43]. These findings underscore the substantial impact of socioeconomic deprivation on the outcomes of patients with CRC, primarily due to its influence on the timing of diagnosis.

Our analysis revealed a significant correlation between socioeconomic deprivation and CRC survival probabilities. The cohort with the lowest level of deprivation exhibited a 5.6% higher survival probability than the most deprived group (Table 1). Furthermore, in our comprehensive model incorporating both individual and regional variables, we observed that each unit increase in the DI corresponded to a 6.6% decrease in survival probability. These findings align with those of previous research demonstrating an inverse relationship between socioeconomic status and cancer survival [44]. Our study corroborates this association and quantitatively measures its magnitude, offering valuable insights for healthcare policy formulation and resource allocation.

Our analysis indicated a substantial improvement in survival probability between 2003 and 2020, with a generally positive trend in recent years. This observation corroborates the findings of a previous study that examined the KCCR data from 1996 to 2015 and reported increasing survival probabilities over time. Prior research that emphasized tumor location noted a particularly significant enhancement in the survival probabilities of patients with rectal cancer. These improvements are largely attributable to advancements in surgical techniques and chemotherapy and radiotherapy methodologies [45]. A systematic review highlighted the significant disparities in CRC treatment based on socioeconomic factors. Patients in more deprived areas have reduced access to chemotherapy, and there is evidence that inequalities in surgical interventions are correlated with deprivation levels [46]. These findings are consistent with our observations. As illustrated in Figure 2, the survival graphs for the distant stage exhibited steeper slopes than those of other stages, indicating that the negative impact of deprivation on survival probability was particularly pronounced in the distant stage rather than in the local or regional stages. Furthermore, when comparing the 1-year, 3-year, and 5-year survival graphs, the 5-year survival graph showed the steepest slope, indicating that patients from more deprived backgrounds had progressively lower survival probabilities over longer disease courses than did less-deprived patients. The primary distinction between treatment protocols for early- and advanced-stage CRC lies in the application of chemotherapy, particularly in Stage IV cases. The requirement for prolonged and consistent chemotherapy regimens can have substantial implications on patients’ professional and economic activities, potentially posing challenges to treatment adherence and continuity of care. Furthermore, patients with socioeconomically deprived backgrounds often present with other underlying diseases [47]. The presence of severe underlying conditions may compromise a patient’s overall health status, potentially limiting their eligibility for chemotherapy [48,49]. Notably, a recent study analyzing 280,543 patients across 19 cancer types found that patients with comorbidities were less likely to receive chemotherapy or multimodality treatment [49].

Our findings indicate that enhancing chemotherapy accessibility in socioeconomically disadvantaged groups is crucial in improving survival outcomes. This suggests that various measures to improve access and support CRC treatment for patients in deprived areas should be implemented nationally [50]. Without the NCSP and NHIS’s additional control efforts for the social determinants of health, the Universal Health Coverage strategy for cancer in Korea could lead to an unintended widening of health inequalities [51,52,53]. It is essential to recognize that chemotherapy is not the only factor contributing to these outcomes. Additional research is necessary to ascertain whether the significant negative impact of deprivation on patients with advanced-stage disease is a common phenomenon across various cancer types or is specific to CRC. Such research will yield valuable data for shaping comprehensive national healthcare strategies.

Analysis of survival probability between 2003 and 2020 revealed a general upward trend. However, the 2012–2018 period exhibited minor fluctuations. This trend resumed in 2018. Nonetheless, the precise cause for this pattern remains unclear. CRC treatment encompasses a complex interplay of factors, including surgical interventions, chemotherapy protocols, and radiation therapy regimens. This multifaceted nature precludes attributing observed effects to any single factor without substantial empirical evidence. Although the Korean Ministry of Food and Drug Safety approved regorafenib and trifluridine–tipiracil in 2013 and 2019, respectively, there is currently insufficient data to conclusively determine their isolated impact on survival probabilities across large patient cohorts. In Korea, regorafenib and trifluridine–tipiracil are not covered by the NHIS, making it challenging for deprived patients to access these treatments. This lack of coverage may be one of the reasons why deprivation has a more pronounced impact on survival in distant-stage cases. Additional research is warranted to provide more comprehensive insights into and interpretations of these trends.

Based on our study, we can propose strategies to improve CRC screening methods and survival probabilities. Our research clearly demonstrated the negative impact of socioeconomic deprivation on CRC survival, with a particularly pronounced effect in advanced stages. This highlights the urgent need for targeted policy support to enhance access to screening and treatment in more deprived areas. To achieve this, it is crucial to assess the level of deprivation in each region first. Specifically, analyzing deprivation at the small-area level allows for more precise identification of regions requiring efforts to reduce health inequalities. Each country should utilize a small-area level deprivation index to identify vulnerable areas and implement focused screening programs and educational initiatives in these regions. Additionally, to increase screening participation rates, community-based awareness programs should be combined with practical measures such as financial support.

Our study has limitations. Owing to the use of large national registry data and ICD-10 codes, there were limitations in understanding information beyond what was registered in the registry. For instance, individual-level information, such as family history, radiation treatment history, diet, smoking, drinking, chronic diseases, and body mass index, is missing. This is an inherent limitation of large registry data that cannot be easily overcome. However, using such large-scale data ensures the reliability of the analytical results. Second, as this analysis was applied to small-area units in Busan, an urban–rural complex region that includes rural areas, it cannot be applied to the overall pattern of Korean society and the extent of its disparities. Follow-up research using the entire national cancer registry data is necessary. Third, the DI was calculated using 2015 data and applied with the same value for 2003–2020. Owing to limitations in the available data, it is difficult to fully capture the changing patterns of each region related to year-by-year changes. However, because regional characteristics are historically and socioeconomically structured and do not change significantly from year to year, we believe that the 2015 DI, selected considering the lag period, is the best choice. Despite these limitations, we believe that the analysis results of long-term changes and regional disparity patterns at the smallest administrative district level can have significant policy implications.

## 5. Conclusions

This study identified the effects of socioeconomic deprivation on CRC. The results showed that, as deprivation increased, survival probability tended to decrease. The negative effect of deprivation on survival was significantly stronger at the distant stage than at the locoregional stage. Additionally, more deprived patients had a lower probability of survival than less deprived patients as the disease duration increased. This indicates that, despite Korea’s active nationwide healthcare benefits provided through the K-NHIS, inequalities remain. This suggests that various socioeconomic measures should be implemented nationally to improve access to CRC treatment for patients in deprived areas.

## Figures and Tables

**Table 1:** The median survival probability of colorectal cancer according to study characteristics.

		Number of Patients with Cancer		Median Survival	
		(%)	*p*-Value	Probability (%)	*p*-Value
Age group	0–44	2243 (6.4)	<0.001	85.4 (83.8, 86.9)	<0.001
	45–64	15,577 (44.5)		83.7 (83.1, 84.4)	
	65–74	10,347 (29.6)		78.4 (77.5, 79.2)	
	≥75	6832 (19.5)		59.4 (58.1, 60.7)	
Gender	Men	21,006 (60.0)	<0.001	78.6 (78.0, 79.1)	<0.001
	Women	13,993 (40.0)		76.5 (75.7, 77.2)	
Stage	Local	12,967 (37.0)	<0.001	92.3 (91.8, 92.8)	<0.001
	Regional	15,872 (45.3)		82.8 (82.2, 83.4)	
	Distant	6160 (17.6)		31.5 (30.3, 32.8)	
Year of diagnosis	2003	828 (2.4)	<0.001	68.0 (64.7, 71.4)	<0.001
	2004	959 (2.7)		70.5 (67.5, 73.6)	
	2005	1165 (3.3)		72.6 (70.0, 75.4)	
	2006	1295 (3.7)		75.5 (73.1, 78.0)	
	2007	1547 (4.4)		76.5 (74.3, 78.8)	
	2008	1688 (4.8)		74.7 (72.5, 76.9)	
	2009	1953 (5.6)		75.9 (74.0, 78.0)	
	2010	2152 (6.1)		77.8 (75.9, 79.6)	
	2011	2267 (6.5)		79.5 (77.7, 81.3)	
	2012	2423 (6.9)		81.3 (79.7, 83.0)	
	2013	2439 (7.0)		79.1 (77.4, 80.9)	
	2014	2352 (6.7)		76.9 (75.1, 78.8)	
	2015	2380 (6.8)		76.5 (74.7, 78.3)	
	2016	2354 (6.7)		77.6 (75.8, 79.4)	
	2017	2436 (7.0)		79.7 (78.0, 81.4)	
	2018	2309 (6.6)		75.9 (74.1, 77.8)	
	2019	2273 (6.5)		80.7 (79.0, 82.4)	
	2020	2179 (6.2)		87.5 (86.1, 89.0)	
Deprivation index	1st (least)	7180 (20.5)	<0.001	80.5 (79.6, 81.5)	<0.001
	2nd	7057 (20.2)		78.1 (77.1, 79.2)	
	3rd	6762 (19.3)		77.2 (76.2, 78.3)	
	4th	7263 (20.8)		77.6 (76.5, 78.6)	
	5th (most)	6737 (19.2)		74.9 (73.8, 76.1)	

Median survival time of colorectal cancer: 157 weeks.

**Table 2:** Parameter estimates from frailty survival model (OR, 95%CI).

		Model 0	Model 1		Model 2	
		HR (95%CI)	HR (95%CI)		HR (95%CI)	
Fixed effect						
Individual level						
Age group (years)	0–44		(Ref.)		(Ref.)	
	45–64		1.148 (1.027–1.284)	*	1.141 (1.021–1.276)	*
	65–74		1.657 (1.48–1.856)	***	1.638 (1.462–1.834)	***
	≥75		3.936 (3.514–4.408)	***	3.903 (3.485–4.371)	***
Gender	Men		(Ref.)		(Ref.)	
	Women		0.973 (0.929–1.018)		0.973 (0.929–1.019)	
Stage	Local		(Ref.)		(Ref.)	
	Regional		2.328 (2.168–2.499)	***	2.325 (2.166–2.497)	***
	Metastasis		15.11 (14.09–16.21)	***	15.09 (14.07–16.19)	***
Year of diagnosis						
	2003		1.500 (1.291–1.743)		1.491 (1.284–1.732)	***
	2004		1.235 (1.066–1.431)	***	1.237 (1.068–1.433)	**
	2005		1.202 (1.045–1.382)	**	1.202 (1.045–1.382)	**
	2006		1.084 (0.942–1.247)	***	1.087 (0.945–1.250)	
	2007		1.004 (0.878–1.147)		1.004 (0.879–1.148)	
	2008		1.031 (0.905–1.175)		1.029 (0.903–1.172)	
	2009		1.043 (0.92–1.182)		1.044 (0.921–1.183)	
	2010		(Ref.)		(Ref.)	
	2011		0.913 (0.805–1.036)		0.916 (0.807–1.039)	
	2012		0.776 (0.683–0.881)		0.776 (0.683–0.881)	***
	2013		0.883 (0.779–1.001)	***	0.883 (0.779–1.001)	*
	2014		0.966 (0.855–1.092)	*	0.966 (0.855–1.092)	
	2015		0.864 (0.764–0.977)		0.864 (0.764–0.978)	*
	2016		0.796 (0.703–0.902)	*	0.795 (0.702–0.900)	***
	2017		0.727 (0.64–0.825)	***	0.726 (0.639–0.824)	***
	2018		0.791 (0.698–0.896)	***	0.792 (0.699–0.898)	***
	2019		0.624 (0.545–0.715)	***	0.625 (0.546–0.715)	***
	2020		0.491 (0.420–0.575)	***	0.490 (0.419–0.574)	***
Area level						
Deprivation					1.066 (1.037–1.096)	***
Random part						
σ(95% CI)		0.126 (0.097–0.165)	0.119 (0.090–0.157)		0.111 (0.082–0.148)	
MHR		1.128 (1.097–1.170)	1.120 (1.089–1.162)		1.111 (1.082–1.152)	

*: <0.05, **: <0.01, ***: < 0.001. CI, confidence interval; MHR, median hazard ratio; OR, odds ratio.

**Figure A1:**
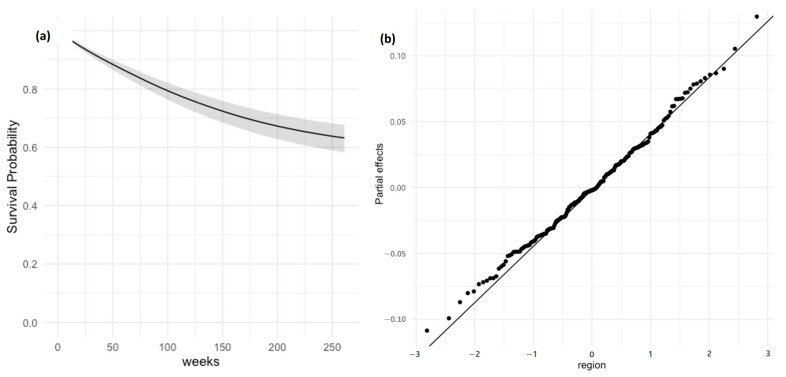
Results from multilevel survival analysis in Model 2: (**a**) survival curve by specific variables (Age group: 65–74 years, Gender: Men, Stage: regional, Year of diagnosis: 2018, Deprivation index: 0) and (**b**) demographic regional differences in 5-year survival rates.

## Data Availability

The datasets presented in this article are not readily available because the data were collected or recorded in a way that protects the privacy of individual participants in accordance with the Korean government’s Cancer Control Act. However, de-identified sample data can be made available upon request from the corresponding author.

## Abbreviations

Confidence interval, CI; colorectal cancer, CRC; deprivation index, DI; early-onset colorectal cancer, EOCRC; geographic information system, GIS; German Index of Socioeconomic Deprivation, GISD; hazard ratio, HR; Institutional Review Board, IRB; International Classification of Diseases 10th Revision, ICD-10; Korea Central Cancer Registry, KCCR; Korean National Health Insurance Service, K-NHIS; median hazard ratio, MHR; National Cancer Screening Program, NCSP; not significant, NS; odds ratio, OR.
